# Supplementary material for: Real-Life Use Patterns of Androgen Receptor Pathway Inhibitors (ARPIs): A Nationwide Register-Based Study in Finland During 2012–2023
Source: Cancers (Basel). 2025 Sep 29;17(19):3162. doi: 10.3390/cancers17193162 (PMC12524319; doi:10.3390/cancers17193162)
Supplement: Supplementary file 1 [file cancers-17-03162-s001.zip › cancers-3754135-supplementary.pdf]

Supplementary tables S1 and S2

Supplementary Table S1. Reimbursement history of novel androgen receptor pathway inhibitors (ARPIs) in Finland.

**Year**	**Change in the reimbursement of ARPIs in the treatment of prostate cancer**<br>**(The Anatomical Therapeutic Chemical (ATC) classification code of the medicine)**
2012	**Abiraterone (ATC-code L02BX03)** was included in the Finnish national health insurance (NHI) in September 2012, with reimbursement limited to the treatment of Metastatic Castration-Resistant Prostate Cancer (mCRPC) for patients whose prostate cancer progressed during or after docetaxel-based chemotherapy.
2014	**Enzalutamide (L02BB04)** was included in the NHI in September 2014, with reimbursement limited to the treatment of mCRPC for patients whose prostate cancer progressed during or after docetaxel-based chemotherapy.
2018	In 2018, a financial managed entry agreement was made for both **abiraterone** and **enzalutamide**, extending their reimbursement to treat asymptomatic or mildly symptomatic mCRPC patients after failure of androgen deprivation therapy (ADT), where chemotherapy is not yet clinically indicated.
2020	**Apalutamide (L02BB05)** and **darolutamide (L02BB06)** were included into the NHI in August 2020 and October 2020, respectively. Their reimbursement was limited to non-metastatic castration resistant prostate cancer (nmCRPC) patients who are at high risk of developing metastatic disease. A financial managed entry agreement was also made.
2021	In December 2021, the reimbursement of **darolutamide** was limited to the treatment of metastatic hormone-sensitive prostate cancer (mHSPC) in combination with ADT. A financial managed entry agreement was also made.
2022	In May 2022, the reimbursement of **apalutamide** was extended to include the treatment of men with mHSPC in combination with ADT. Additionally, a financial managed entry agreement was established.<br><br>**Abiraterone** was included in the reference price system in October 2022.
2023	The reimbursement of **enzalutamide** was extended to the treatment of hormone-sensitive prostate cancer in March 2023.

Supplementary Table S2. Calculation of costs for sequential use.

Medicine, number of sequential users	Median duration in sequential use, days divided by the number of needed packages (of 28 days) for the needed treatment period	Cost of one package * (retail price including VAT) (Hila lists 2022; 2023)	Total costs for all sequential users (calculated as number of users* median number of packages* price of a package)
Abiraterone<br><br>(n= 1,354)	182 / = 6.5 packages	3,388 / 98 €	20,966,764 €
Enzalutamide<br><br>(n=1,321)	195/7 packages	2,502 €	22,610,574 €
Apalutamide (n=5)	106 / 4 packages	2,781 €	55,619 €
Darolutamide (n=5)	342 = 12 packages	2,781 €	166,858 €
Total			**43,799,815 €**<br><br>**43.8 M€**

* For abiraterone, 75% of its use was expected to occur before the introduction of generic products, while 25% of its use was expected to be generic, as abiraterone was included in the reference price system in October 2022.
